# Molecular constraints on tolerance‐resistance trade‐offs: Is there a cost?

**DOI:** 10.1002/pei3.10125

**Published:** 2023-10-25

**Authors:** J. Miles Mesa, Ken N. Paige

**Affiliations:** ^1^ Department of Evolution, Ecology and Behavior University of Illinois at Urbana–Champaign Urbana Illinois USA

**Keywords:** Arabidopsis, endoreduplication, glucosinolates, overcompensation, oxidative pentose phosphate pathway, resistance‐tolerance tradeoffs, shikimate acid pathway

## Abstract

Plants possess myriad defenses against their herbivores, including constitutive and inducible chemical compounds and regrowth strategies known as tolerance. Recent studies have shown that plant tolerance and resistance are positively associated given they are co‐localized in the same molecular pathway, the oxidative pentose phosphate pathway. However, given that both defensive strategies utilize carbon skeletons from a shared resource pool in the oxidative pentose phosphate pathway there are likely costs in maintaining both resistance‐tolerance strategies. Here we investigate fitness costs in maintaining both strategies by utilizing a double knockout of *cyp79B2* and *cyp79B3*, key enzymes in the biosynthetic process of indole glucosinolates, which convert tryptophan to indole‐3‐acetaldoxime (IAOx) and is further used to produce indole glucosinolates. These mutant plants are devoid of any indole glucosinolates thus reducing plant resistance. Results show that knocking out indole glucosinolate production and thus one of the resistance pathways leads to an approximate 94% increase in fitness compensation shifting the undercompensating wild‐type Columbia‐0 to an overcompensating genotype following damage. We discuss the potential mechanistic basis for the observed patterns.

## Graphical abstract

Recent studies have shown that plant tolerance and resistance are positively associated given they are co‐localized in the same molecular pathway, the oxidative pentose phosphate pathway. However, given that both defensive strategies utilize carbon skeletons from a shared resource pool in the oxidative pentose phosphate pathway there are likely costs in maintaining both resistance‐tolerance strategies. Results show that knocking out indole glucosinolate production and thus one of the resistance pathways leads to an approximate 94% increase in fitness compensation shifting the undercompensating wild‐type Columbia‐0 to an overcompensating genotype following damage.

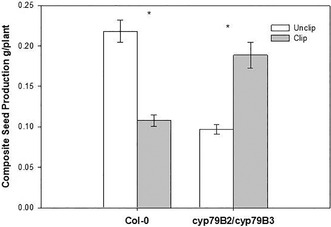

## 1 INTRODUCTION

Plants possess multiple types of defenses against herbivores, including constitutive and inducible chemical compounds, structural traits (collectively, resistance) and regrowth/fitness strategies (tolerance). These defense strategies typically co‐occur within the same plant (Koricheva et al., 2004). Multiple defense mechanisms have been hypothesized to be costly for a plant, as investment in one anti‐herbivore defense is assumed to reduce resources available for other defenses and growth and reproduction (Strauss et al., 2002). Therefore, constraints on multiple defensive strategies due to resource allocation costs are predicted to manifest as a negative association between the defenses, however, this is not always the case (e.g., see Zust & Agrawal, 2017). Such a trade‐off has been predicted to occur between plant tolerance (fitness compensation) and resistance chemistry (van der Meijden et al., 1988). If a trade‐off were to exist it would have important evolutionary ramifications because it would result in selection leading either to maximal resistance or maximal tolerance, but not both. However, a meta‐analysis on plant resistance and tolerance showed most natural populations appear to be comprised of a mixture of both strategies (e.g., see Leimu & Koricheva, 2006), possibly due to selection for the maintenance of both traits caused by the joint feeding of both generalist and specialist herbivores.

What has been missing from studies of potential trade‐offs between tolerance and resistance is an understanding of the molecular genetic pathway involved in tolerance and its relationship to well‐characterized molecular pathways involved in chemical defense, such as the shikimate pathway (Mesa et al., 2017). Our recent studies on the molecular underpinnings of tolerance (with an emphasis on the phenomenon of overcompensation) have shown that plant tolerance and resistance are positively correlated due to a sharing of the same molecular genetic pathways (e.g., see Figure S1), explaining in part the inability to uncover a physiological trade‐off between these two strategies (Mesa et al., 2017; Siddappaji et al., 2013), yet acknowledging that trade‐offs could still occur at lower levels of the trait hierarchy and manifest positively at higher levels (Agrawal et al., 2010). Specifically, we have uncovered a gene, glucose‐6‐phosphate‐1‐dehydrogenase (G6PD1, At5g35790.1), which plays a major role in controlling the compensatory response in *Arabidopsis thaliana* following the removal of the plant's primary inflorescence (Siddappaji et al., 2013). G6PD1 is the central regulatory enzyme in the oxidative pentose phosphate (OPP) pathway that plays a key role in plant metabolism generating NADPH and a variety of metabolic intermediates for biosynthetic processes including resistance to oxidative damage, the production of plant secondary defensive compounds, such as glucosinolates (of interest herein) and the production of ribo‐ and deoxyribonucleotides (Maeda & Dudareva, 2012).

We have also shown that following the removal of apical dominance, phenotypically plastic increases in ploidy level via endoreduplication leads to rapid regrowth and an increase in fitness, explaining, in part, the phenomenon of overcompensation in plants (Scholes & Paige, 2014). Endoreduplication is the replication of the genome without mitosis, which leads to endopolyploidy, an increase in cellular chromosome number (e.g., see Nagl, 1976). Removal of the apical meristem by herbivores eliminates production of the plant hormone auxin, leading to a rapid break in dormancy of axillary buds and subsequent stem elongation. High levels of auxin are also known to repress the endocycle, and by contrast, lower levels of auxin trigger an exit from mitotic cycles and an entry into endocycles (Ishida et al., 2010). Insect leaf‐feeding also can trigger endoreduplication by the upregulation of jasmonic acid, which also lowers auxin production and can lead to overcompensation in some ecotypes of *Arabidopsis* (Mesa et al., 2019). Thus, there is a direct link between endoreduplication and plant damage.

Increasing chromosome number through endoreduplication and therefore gene copy number provides a means of increasing expression of vital genes (e.g., see Bourdan et al., 2012) (such as G6PD1) or genetic pathways that promote rapid regrowth rates following herbivory. G6PD1 feeds compounds into the OPP pathway for nucleotide biosynthesis, by the provision of ribose‐5‐phosphate, necessary for the significant increase in chromosome number via endoreduplication. The increase in DNA content then feeds back positively on pathways involved in metabolism (e.g., G6PD1) and defense (e.g., glucosinolate production) through increased gene expression (more copies due to increases in endoreduplication following damage. An increase in total cellular DNA content through endoreduplication also leads to extensive cell growth via cell expansion (Melaragno et al., 1993). Rapid growth and development following the removal of apical dominance may be enhanced by maximizing nutrient transport (with fewer plasmodesmata), protein synthesis (with more copies of DNA), photosynthetic rates (with an increase in the number of chloroplasts) and light and water absorption (with larger cell size and storage capacity) (Lee et al., 2009). Importantly, the experimental overexpression of ILP1 (Increased Level of Polyploidy1), an endoreduplication enhancer, increases glucosinolate production and compensation (from undercompensation to overcompensation) in a genotype of *A. thaliana* that typically suffers reduced fitness when damaged (Mesa et al., 2017; Scholes & Paige, 2014), demonstrating a causal relationship between the process of endoreduplication, fitness compensation (tolerance), and chemical defense (see Figure S2).

Overall, these results translate into a positive relationship between plant tolerance and resistance (Mesa et al., 2017). Despite the molecular constraints that lead to a positive association between tolerance and resistance, the lack of a negative correlation does not preclude evidence of a trade‐off given the costs in maintaining both strategies, as both strategies utilize carbon skeletons from a shared resource pool in the OPP pathway. Here, we specifically assess the fitness cost of resistance in *A. thaliana* by utilizing a double knockout mutant for two cytochrome P450s, key enzymes in the biosynthetic process of indole glucosinolates, which convert tryptophan to indole‐3‐acetaldoxime (IAOx); IAOx is then further used in the production of indole glucosinolates (Bender & Celenza, 2009). Specifically, we assessed whether knocking out one of two glucosinolate pathways, the indole glucosinolate pathway (as opposed to the aliphatic glucosinolate pathway) and thus lowering resistance leads to an increase in fitness compensation in the undercompensating wild‐type Columbia‐0 following the removal of the apical meristem (simulating mammalian herbivory). Overall, results show a significant cost of plant resistance with Columbia‐0 shifting from an undercompensating ecotype to an overcompensating ecotype.

## 2 STUDY SYSTEM

### 2.1 Species

*Arabidopsis thaliana*, mouse‐ear cress, is a small, mostly selfing plant in the Brassicaceae family. While native to Europe, *A. thaliana* has a wide geographical range spanning Eurasia, North Africa and North America. Typically, *A. thaliana* is found as a winter annual where seeds of *A. thaliana* germinate in the fall after passing the summer in a dormant state and grow into an overwintering rosette, and following stem elongation in the spring, produce flowers that develop into seed pods known as siliques. *A. thaliana* is fed upon by a variety of species including flea beetles, aphids, leaf miners, caterpillars, deer and rabbits. *A. thaliana* thus frequently experiences leaf and apical meristem damage and has a suite of resistance characters such as trichomes, proteinase inhibitors and glucosinolates that deter and inhibit feeding by herbivores and a suite of tolerance strategies ranging from undercompensation to overcompensation (e.g., Siddappaji et al., 2013).

### 2.2 Glucosinolates

Glucosinolates constitute a large and diverse group of defensive secondary metabolites characteristic of the order Brassicales, which includes *A. thaliana*, our organism of study. Glucosinolates (mustard oil glucosides) are nitrogen and sulfur rich natural plant secondary products that consist of a sulfonated oxime and a β‐thioglucose moiety, but differ in side chain structures (Pfalz et al., 2009). There have been ~40 glucosinolates found in *Arabidopsis*, out of the 120 glucosinolates identified, most of which are classified into three subgroups based on the biosynthetic amino acid precursor, those subgroups being indole, aliphatic and benzenic. Indole and aliphatic glucosinolates constitute most of the diversity of glucosinolates in *A. thaliana* (Brown et al., 2003). Indole glucosinolates, our chemicals of interest, are composed of four individual compounds: glucobrassicin, 4‐methoxy‐glucobrassicin, neoglucobrassicin and 4‐hydroxyglucobrassicin (Brown et al., 2003).

Many studies have shown that glucosinolate breakdown products deter generalist and specialist herbivores on *A. thaliana* (e.g., Agrawal & Kurashige, 2003). Upon herbivory, glucosinolates stored in the vacuole are mixed with the enzyme myrosinase (known as the “mustard bomb”), a β‐thioglucosidase that is separated in scattered specialist cells known as myrosin cells. Myrosinase cleaves the β‐glucose moiety from glucosinolates, leading to a variety of toxic breakdown products, such as bioactive nitriles, epithionitriles and isothiocyanates based on reaction conditions and protein factors such as epithiospecifier protein (Zhang et al., 2006).

In *A. thaliana*, indole glucosinolate synthesis involves a catalyzed conversion of tryptophan to indole‐3‐acetaldoxime (IAOx) carried out by two cytochrome P450s, *cyp79B2* and *cyp79B3* (Hull et al., 2000). IAOx is further catalyzed through four subsequent reactions to form glucobrassicin, the most abundant indole glucosinolate found in *A. thaliana*. Glucobrassicin can then be further modified to the other three indole glucosinolates found in *A. thaliana* with modified indole rings (Bender & Celenza, 2009).

## 3 METHODS

### 3.1 Costs of resistance

To assess costs between plant tolerance and resistance, 100 seeds of Col‐0 and 100 seeds of *cyp79B2 cyp79B3* double mutant lines were planted and grown. While single cyp mutant lines show little deficiency in the ability to produce indole glucosinolates, double mutants are completely devoid of any indole glucosinolates (Zhao et al., 2002). The *cyp79B2 cyp79B3* double mutant line was kindly obtained from the laboratory of John Celenza at Boston University (Department of Biology, Boston, Massachusetts). Of particular note, wildtype Col‐0 on average produces a high concentration of indole glucosinolates when compared to other *A. thaliana* accessions (Kliebenstein et al., 2001).

The *cyp79B2 cyp79B3* double mutant was uncovered from T‐DNA insertion lines of *cyp79B2* and *cyp79B3* in the Col‐0 background, identified from the Salk Institute collection of T‐DNA insertion lines by PCR. Primers 79B2‐5P (5′‐TGGACAAGTATCATGACCCAATC ATCCACG‐3′) and LB (5′‐GGCAATCAGCTGTTGCCCGTCTCACTGGTG‐3′) were used to identify a T‐DNA insertion at 1512 bp after the ATG of the *cyp79B2* gene. The insertion is in the second exon of the *cyp79B2* gene. Primer 79B3‐5P (5′‐ TGTTCTATGCATGGACTGGT GGTCAACATG‐3′) and LB were used to identify a T‐DNA insertion at 1425 bp after the ATG site of the *cyp79B3* gene. The insertion is in the intron between the two exons of the *cyp79B3* gene. The T‐DNA insertion in the *cyp79B3* gene is a tandem T‐DNA insertion with LB flanking sequences at both ends of the T‐DNA insertion. The insertions were confirmed by DNA sequencing of the PCR fragments generated with the LB primer and the gene specific primers (Zhao et al., 2002).

*Arabidopsis* lines were grown in a greenhouse on the campus of the University of Illinois, Champaign, under 12 h of light (~100 uE/M^2^/s) and dark. Plants were grown individually in 3.5‐inch pots in L1 Sunshine mix. Seeds/seedlings were kept moist during germination, and plants were then watered daily to maintain soil moisture without saturating the soil. Plants were not fertilized. When inflorescences reached 6 cm, about 3.5 weeks, 50 plants of each ecotype were randomly clipped, leaving approximately 1 cm of inflorescence (comparable to natural mammalian herbivory (Siddappaji et al., 2013)).

At 6.5 weeks, 30 plants of Col‐0 (15 clipped, 15 unclipped) were analyzed for indole glucosinolate concentration. Inflorescence material was taken from both clipped and unclipped plants. In addition, the level of indole glucosinolates were assessed from 30 plant (15 clipped and 15 unclipped) samples of the *cyp79B2 cyp79B3* double mutant line to verify that there were in fact, undetectable levels of indole glucosinolates, consistent with the findings of Zhao et al., 2002. All samples were frozen in liquid nitrogen and stored at –80°C prior to freeze‐drying. Freeze‐dried tissues were ground into a fine powder and stored at –20°C prior to glucosinolate analysis. Glucosinolates were extracted from finely ground freeze‐dried tissue, converted to desulphoglucosinolates with arylsulfatase and analyzed via high pressure liquid chromatography (HPLC). Freeze‐dried powder, 50 mg, and 0.5 mL of 70% methanol were added to 2.5 mL tubes and placed on a heating block at 95°C for 10 min, mixing frequently. Samples were cooled on ice and 0.125 mL glucosinolabin was used as an internal standard and centrifuged at 3000×g for 10 min. The supernatant was saved and the pellet was re‐extracted with another 0.5 mL 70% methanol at 95°C for 10 min and the two extracts were combined. Protein was subsequently precipitated with 0.15 mL of a 1:1 mixture of 1 M barium acetate and 1 M lead acetate and centrifuged at 12,000×g for 1 min. Each sample was then loaded onto a column containing DEAE Sephadex A‐25 resin for desulfation via arylsulfatase for 18 h and the remaining desulfo‐GS eluted. Desulphoglucosinolates were separated on an HPLC system (Agilent 1100 HPLC system, with a G1311A bin pump, a G1322A vacuum degasser, a G1316A thermostatic column compartment, a G1315B diode array detector and an HP 1100 series G1313A autosampler) with a variable ultraviolet detector set at 229 nm wavelength. Elution of desulphoglucosinolates occurred over 45 min with a linear gradient of 0%–20% acetonitrile in water with a flow rate of 1.0 mL/min. Glucosinolate concentration was established using glucosinalbin as an internal standard, a glucosinolate not found in *A. thaliana*. UV response factors for different glucosinolates were used as determined by Wathelet et al. (2001). Indole glucosinolates were estimated by adding the four indole glucosinolates observed in Col‐0.

Upon plant senescence (8 weeks), remaining plants of both ecotypes were analyzed for fitness (70 plants per ecotype; 35 clipped, 35 unclipped). Fitness measures included the number of siliques, seeds per plant and the average seed weight per plant. Our previous studies have shown that seeds are a good measure of plant fitness as there are no significant differences in germination success between clipped and unclipped plants of *A. thaliana* (e.g., Siddappaji et al., 2013). We measured total silique number for each plant and counted total seed production in 3 randomly selected siliques from each plant. Seeds per silique were averaged per ecotype and treatment then multiplied by total silique number for each plant to obtain seed totals per plant. Average seed weights were measured by weighing 50 seeds per plant from 10 plants per ecotype × treatment group. Each weight measurement was then divided by the number of seeds to yield average seed weight for each ecotype × treatment group. Additionally, we measured rosette diameter at time of senescence for Col‐0 and *cyp79B2 cyp79B3*.

Potential differences in composite seed production were assessed using an analysis of variance and Type III sums of squares with two treatment factors (genotype and clipping). Rosette diameter was used as a covariate to adjust for differences in plant size. In addition, we regressed total seed production on rosette diameter of *A. thaliana* for both Col‐0 and *cyp79B2 cyp79B3* double mutant lines to justify the use of rosette diameter as an appropriate covariate in the model (see Figure S3). Differences among treatments within genotypes were determined using Tukey pairwise comparisons. Glucosinolate production, between clipped and unclipped Col‐0 plants, was assessed using a two‐sample *t*‐test. Statistical analyses were conducted in Systat (version 13; Systat).

## 4 RESULTS AND DISCUSSION

**FIGURE 1:**
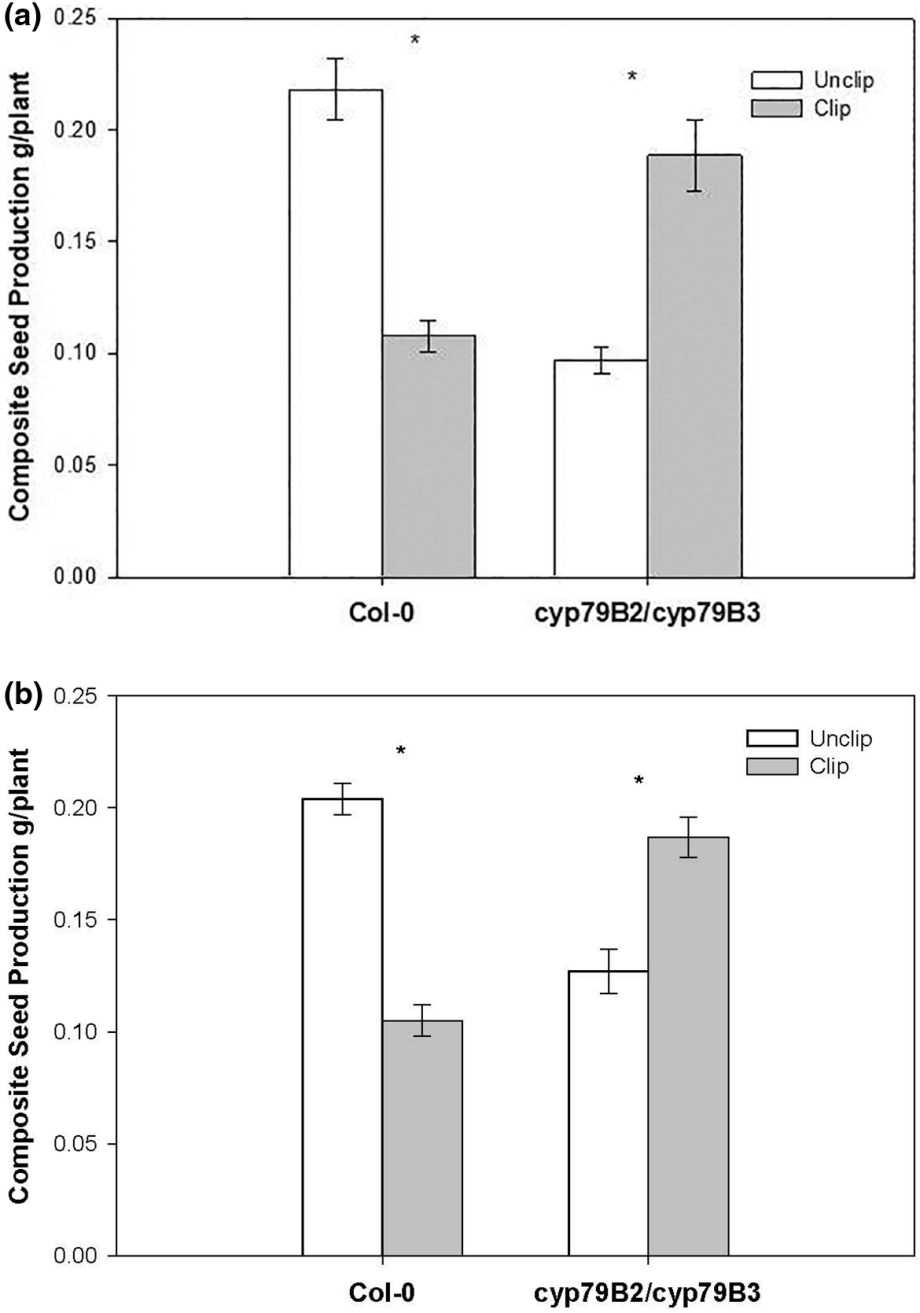
(a) Composite seed production for clipped and unclipped plants for Col‐0 and *cyp79B2 cyp79B3* double mutant lines. Shown are means ± 1 SE. Asterisks indicate significance at a familywise error rate of α = .05 for clipped and unclipped plants. (b) Composite seed production for clipped and unclipped plants for Col‐0 and cyp79B2 cyp79B3 double mutant lines adjusted for rosette size as a covariate. Shown are means ±1 SE. Asterisks indicate significance at a familywise error rate of α = .05 for clipped and unclipped plants.

Genotypes differed in composite seed production in their response to simulated mammalian herbivory (*p* < .001) (Table S1). Specifically, a post hoc Tukey test revealed Col‐0 significantly undercompensated with a 50.5% decrease in composite seed yield (grams/plant) (when comparing damaged plants to undamaged controls, *p* < .001). In contrast, the *cyp79B2 cyp79B3* double mutant with severely impaired resistance significantly overcompensated (*p* < .001) following damage with a 94% increase in composite seed yield (Figure 1a). Results remained the same following adjustment with rosette size run as a covariate to adjust composite seed yield means (Figure 1b). Results show that there is a linear relationship between composite seed yield and rosette size justifying rosette size as an appropriate covariate in our analysis (Figure S3).

Genotypes also differed in indole glucosinolate concentrations in their response to simulated mammalian herbivory (*p* < .001). Unclipped Col‐0 plants produced on average 1.35 μmol g^−1^ of total indole glucosinolates, whereas clipped Col‐0 displayed a 41.48% decrease (producing 0.79 μmol g^−1^; *t* = 2.88, df = 2.28, *p* < .008) in indole glucosinolates following removal of the apical meristem (Figure S1). We also confirmed that the knockout mutants did not produce any indole glucosinolates as shown by Zhao et al. (2002).

In 1988, van der Meijden et al. proposed that there should be a trade‐off between plant resistance and tolerance given that resources are limited. However, we have recently shown that both resistance and tolerance are positively and causally entwined within the same molecular pathway (the OPP pathway leading directly into the shikimate pathway) (Siddappaji et al., 2013). By measuring glucosinolate levels and seed production (our measure of tolerance) following the removal of apical dominance in *A. thaliana* (ranging the entire spectrum of seed production from undercompensation to equal compensation to overcompensation), we have shown that there is a positive association between tolerance and induced resistance. For example, plants that undercompensated for seed production following the removal of apical dominance also showed a decrease in glucosinolate production, whereas plants that equally compensated for seed production equally compensated for glucosinolate production, and plants that overcompensated for seed production overcompensated for glucosinolate production (see Figure S1; from Mesa et al., 2017). Thus, these results lend support to models predicting that selection favors intermediate levels of both resistance and tolerance (Fornoni et al., 2004; Leimu & Koricheva, 2006), questioning the generality of the assumptions behind the trade‐off hypothesis (see e.g., de Jong & van der Meijden, 2000); i.e., when plants experience herbivory from both generalist herbivores that are susceptible to resistance traits and specialist herbivores that can circumvent resistance traits, selection should favor intermediate levels of tolerance and resistance.

Here we show that despite a positive relationship between tolerance and resistance there is still a predictable cost/trade‐off in maintaining them. Our results show that knocking out the indole glucosinolate biosynthetic pathway via a *cyp79B2 cyp79B3* double mutant in a Col‐0 background results in plants overcompensating after tissue damage, with clipped plants having significantly higher total seed production compared to the undercompensating wild‐type Col‐0. As both resistance and tolerance are controlled via the OPP pathway (Mesa et al., 2017; Scholes & Paige, 2014) knocking out production of indole glucosinolates should allow more resources to be shunted toward tolerance.

However, knockout mutants for indole glucosinolate biosynthesis also have reduced levels of the growth regulator indole‐3‐acetic acid (IAA), as indole‐3‐acetaldoxime (IAOx) is used as an intermediate of both indole glucosinolates and IAA biosynthesis. IAOx is reduced by knocking out the Cytochrome P450 enzymes *cyp79B2* and *cyp79B3*, hence reducing IAA (Zhao et al., 2002). IAA is the main auxin in plants, regulating growth and developmental processes such as cell division and elongation, tissue differentiation, and apical dominance. Effects of the downregulation of auxin can be seen in this experiment as rosette diameters of unclipped plants of *cyp79B2 cyp79B3* were significantly smaller than wild‐type plants. Zhao et al. (2002) found similar results, both rosettes and elongating cotyledons were smaller in the *cyp79B2 cyp79B3* double knockouts and auxin was downregulated.

**FIGURE 2:**
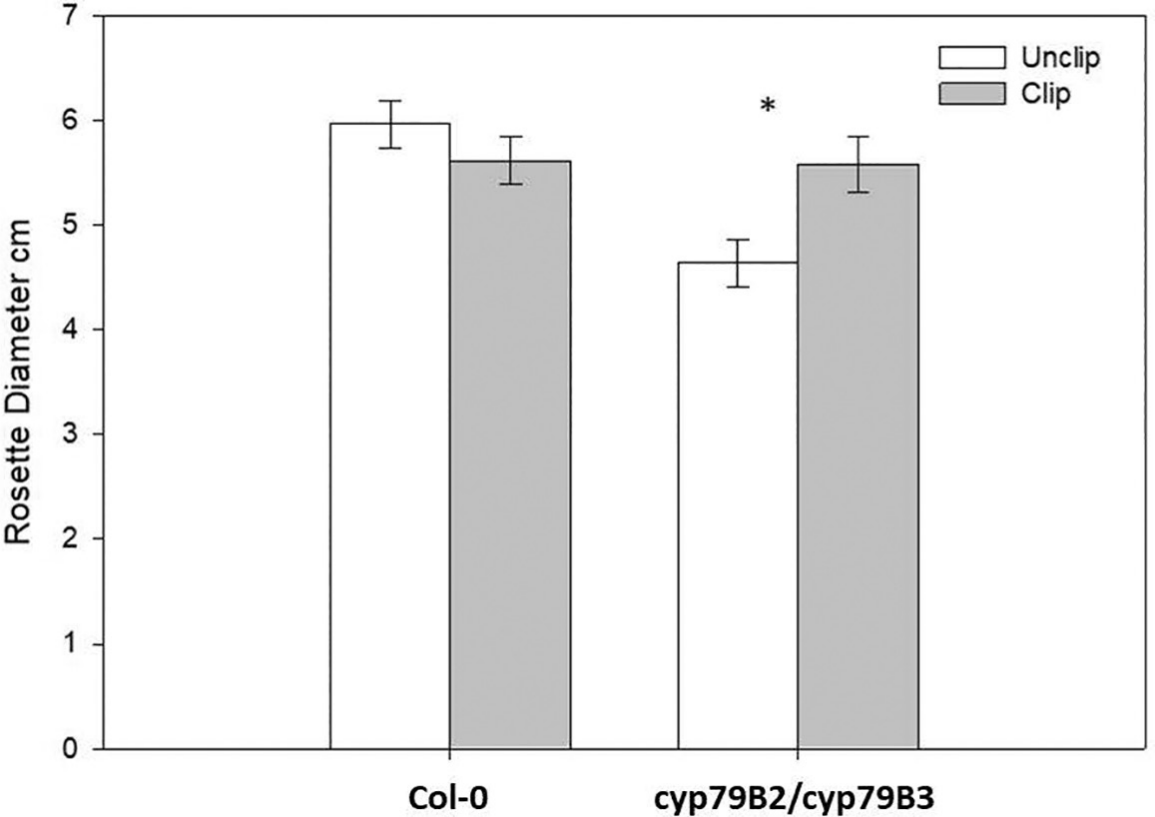
Rosette diameter for clipped and unclipped Col‐0 and cyp79B2 cyp79B3 double mutant lines. cyp79B2 cyp79B3 clipped were significantly larger than unclipped plants. Shown are means ±1 SE. Asterisks indicate significance at a familywise error rate of α = .05.

In contrast, clipped *cyp79B2 cyp79B3* double knockout rosettes were as large as the wild‐type treatments and these plants overcompensated compared to unclipped *cyp79B2 cyp79B3* double knockout plants (Figure 2; note that we found the same statistical outcome in terms of seed production whether we adjusted for unclipped cyp knockout plant sizes or not in this experiment, see Figure 1a,b). It is also clear that the clipped *cyp79B2 cyp79B3* double knockout plants overcompensated when comparing them to the clipped wild‐type plants, consistent with an expected increase in resources toward compensation given that one of two major chemical pathways was knocked out.

A recent review, however, notes that “an emerging consensus suggests that there is close regulatory control of growth and defense by the plant and identifies associations between growth and defense not as the direct result of allocation costs, but rather as prioritization of one process over another. Trade‐offs between growth and defense thus may more often reflect the range of trait combinations that achieve optimal fitness, rather than representing hard physiological limits” (Zust & Agrawal, 2017). Although we agree that regulatory control is essential in growth and defense, the phenomenon of overcompensation (Scholes & Paige, 2014) challenges the notion that growth and defense tradeoffs are primarily/solely regulatory and not the direct result of allocation patterns. This is evidenced by the process of endoreduplication, which leads to overcompensation, an increase in reproductive success. As the number of chromosomes within a cell increase due to endoreduplication, there is a corresponding increase in both chloroplast and mitochondrial numbers (a nucleotypic effect leading to an increase in photosynthetic rates and powering the cells enhanced energy needs), ultimately resulting in an increase in overall above‐ and below‐ground biomass and an increase in fitness (e.g., see Mesa et al., 2017; Scholes & Paige, 2014). Thus, achieving “optimal fitness” primarily as a prioritization process between growth and defense ignores the enhanced resource allocation patterns generated by the phenomenon of endoreduplication/overcompensation (see Figure 1a clipped vs. unclipped *cyp79B2 cyp79B3* double knockout plants). As noted above, this process is initiated by the removal of the apical meristem that eliminates production of the plant hormone auxin, leading to a rapid break in the dormancy of axillary buds and subsequent stem elongation where low levels of auxin trigger an exit from mitotic cycles and an entry into endocycles (Ishida et al., 2010). The increase in chromosome number leads to higher levels of gene expression, regrowth and enhanced fitness (e.g., see Scholes & Paige, 2014).

Furthermore, the combination of clipping the cyp79B2 cyp79B3 knockout, along with the reduction in resistance, likely lead to an increase in IAA production through the process of endoreduplication (via an increase in gene expression; e.g., see Bourdan et al., 2012). Zhao et al. (2002) have shown that alternative pathways of IAA production remain active and these alternative pathways are likely increased through the process of endoreduplication triggered by the removal of apical dominance (Scholes & Paige, 2014), explaining why we do not see the size reductions we see in the unclipped cyp79B2 cyp79B3 knockout (cell cycle values for clipped cyp knockout plants was also significantly higher than for clipped wild‐type plants; 0.500 +/− 0.47 vs. 0.376 +/− 0.057, *t* = 4.29, df = 13, *p* = .001). Thus, the increase in fitness not only involves a reduction in resistance but in this case an increase in the level of IAA through endoreduplication following the removal of apical dominance. It is important to note that the clipped wild‐type plants undercompensated and were not affected by a reduction in IAA, suggesting, at least in part, that it is the reduction in resistance that contributes to the increase in reproductive success.

Mechanistically, bypassing the shikimate pathway, may lead to an increase in the production of glucose‐6‐phosphate‐dehydrogenase‐1 (G6PD1), which plays a key role in compensation and primary metabolism (Siddappaji et al., 2013), ultimately leading to an increase in plant growth and reproduction. There is also the possibility that blockage of the shikimate pathway increases the availability of translational machinery (e.g., ribosomes) leading to an increase in the compensatory response (Caveney et al., 2017) through endoreduplication.

As noted above, pleiotropic effects potentially played a role in reducing rosette size in the unclipped double knockout line, though statistical adjustments for plant rosette size did not change the results of our findings. However, there are further potential pleiotropic effects of altering the indole biosynthetic pathway that may alter flux within the shikimate pathway. For example, IAOx, or their derivatives, produced by *cyp79B2cyp79B3* are responsible for the molecular crosstalk between the glucosinolate and phenylpropanoid pathways (Kim et al., 2020). Additionally, recent studies have shown the importance of endogenous indole glucosinolate levels on tryptophan metabolism (Czerniawski et al., 2022). Therefore, it is not possible to fully disentangle the potential pleiotropic effects from the changes in allocation to defense strategies. In future studies, we suggest that a knockout mutant such as *myb28myb29* within the aliphatic glucosinolate pathway, which is derived from methionine would suffice in avoiding potential pleiotropic antagonistic effects on auxin to further substantiate and clarify our findings (Martínez‐Ballesta et al., 2015).

Despite a positive relationship between tolerance and resistance in *A. thaliana*, our results suggest that plant resistance carries a significant cost in tolerance, suppressing the degree of compensation. Our results are currently unclear as to the mechanistic basis of this resource‐based trade‐off and opens an avenue for further investigation.

What we need to do moving forward, is to address whether endoreduplication is a general mechanism for explaining patterns of tolerance and resistance and tolerance/resistance trade‐offs. We know that endoreduplication is common in plants, with approximately 90% of herbaceous angiosperms being endopolyploid (Nagl, 1976). We also know that there is genetic variation for compensation/tolerance across numerous plant species. For example, within a species some families exhibit overcompensating tolerance, whereas others express incomplete tolerance (e.g., see Siddappaji et al., 2013). What is unknown is the degree to which plant species are genetically/genotypically plastic in terms of the level of endoreduplication following herbivory and more generally how common the association is between endoreduplication, tolerance and plant resistance. Therefore, there is a need for studies on other species to determine whether there is a positive association between the degree of fitness compensation following herbivory, resistance and the level of endopolyploidy, and ultimately, a causal relationship, as we have shown in *A. thaliana*.

## AUTHOR CONTRIBUTIONS

KNP and JMM conceived and designed the experiments. JMM performed the experiments. JMM and KNP analyzed the data. JMM conducted the chemical analyses. JMM and KNP wrote the manuscript.

## CONFLICT OF INTEREST STATEMENT

The authors declare that they have no conflict of interest.

## Supporting information


Data S1.

## Data Availability

Data openly available in a public repository that issues datasets with DOIs. Data are available on Dryad doi:10.5061/dryad.sbcc2frbp.
