# Supplementary material for: Molecular constraints on tolerance‐resistance trade‐offs: Is there a cost?
Source: Plant Environ Interact. 2023 Oct 25;4(6):317–23. doi: 10.1002/pei3.10125 (PMC10711643; doi:10.1002/pei3.10125)
Supplement: Supplementary file 1 — Data S1. [file PEI3-4-317-s001.docx]

**Table S1.** Analysis of Covariance for composite seed yield.

| **Source** | **Type III SS** | **Df** | **Mean Square** | | **F-ratio** | | **p-Value** |
| --- | --- | --- | --- | --- | --- | --- | --- |
| **Rosette** | .306 | 1 | .306 | 137.793 | | .000 | |
| **Genotype** | .000 | 1 | .000 | .116 | | .734 | |
| **Clipping** | .012 | 1 | .012 | 5.343 | | .022 | |
| **Genotype x Clipping** | .191 | 1 | .191 | 85.977 | | .000 | |
| **Error** | .296 | 133 | .002 |  | |  | |

**Figure S1.** Percent differences in composite seed yield and indole glucosinolate production comparing clipped and unclipped plants for 7 *Arabidopsis thaliana* genotypes. Genotypic responses range from undercompensation (negative percent difference values) to overcompensation (positive percent difference values). Asterisks indicate significance at a familywise error rate of α = 0.05 between clipped and unclipped plants. (Figure from Mesa et al. 2017).

**
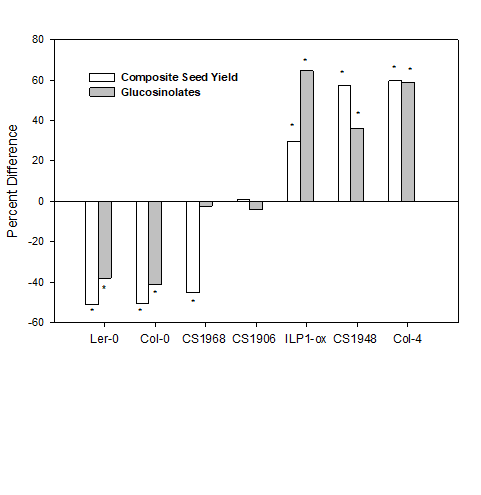
**

**Figure S2.** Percent difference between clipped and unclipped plants for the Col-0 and ILP1-ox mutant. Measures include seeds, glucosinolates and cell cycle values (endopolyploidy). Genotypic responses range from undercompensation (negative percent difference values) to overcompensation (positive percent difference values). Asterisks indicate significance at a familywise error rate of α = 0.05. Plus signs indicate marginal (p < 0.1) significance (Figure from Mesa et al. 2017). Cell cycle values were generated by a separate study (Scholes and Paige 2014).

**
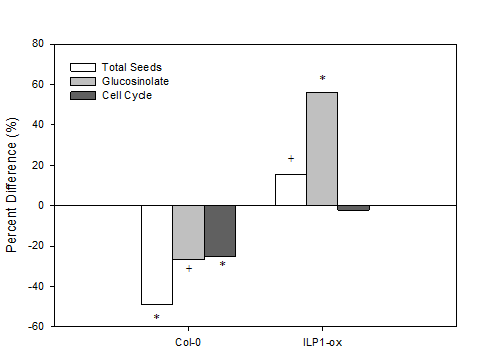
**

**Figure S3.** Regression of rosette diameter and total seeds of *A. thaliana* for both Col-0 and cyp79B2 cyp79B3 double mutant lines. Data show a significant positive relationship between the two variables.

**
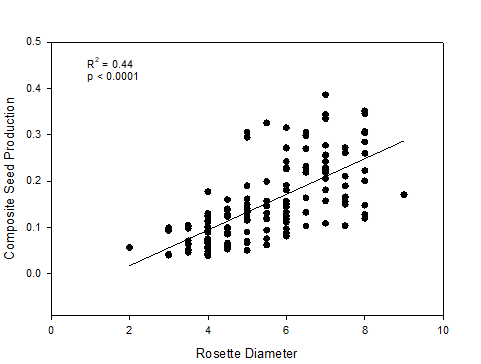
**
